# Supplementary material for: The Role of Intestinal Cytochrome P450s in Vitamin D Metabolism
Source: Biomolecules. 2024 Jun 17;14(6):717. doi: 10.3390/biom14060717 (PMC11201832; doi:10.3390/biom14060717)
Supplement: Supplementary file 1 [file biomolecules-14-00717-s001.zip › biomolecules-(Supplement for publish).pdf]

biomolecules

MDPI

Supplementary Materials:

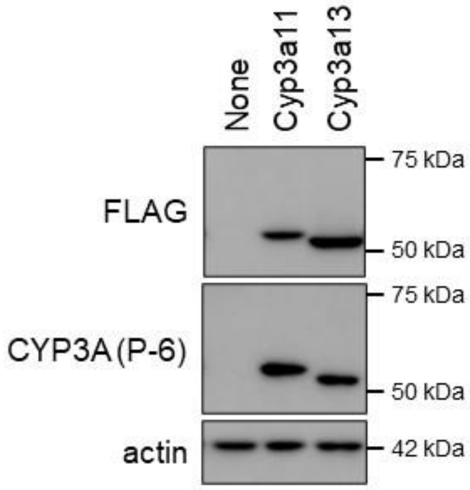

Figure S1. Anti-CYP3A (P-6) recognized mouse Cyp3a11 and Cyp3a13. We constructed a FLAG-tagged Cyp3a11 or Cyp3a13. This FLAG-tagged Cyp3a plasmids were then transfected in HEK293 cells. The total cell lysate (20 μg/lane) was separated by 10% SDS-PAGE gel. Anti-FLAG or CYP3A (P-6) detected both of the Cyp3a11 or Cyp3a13.

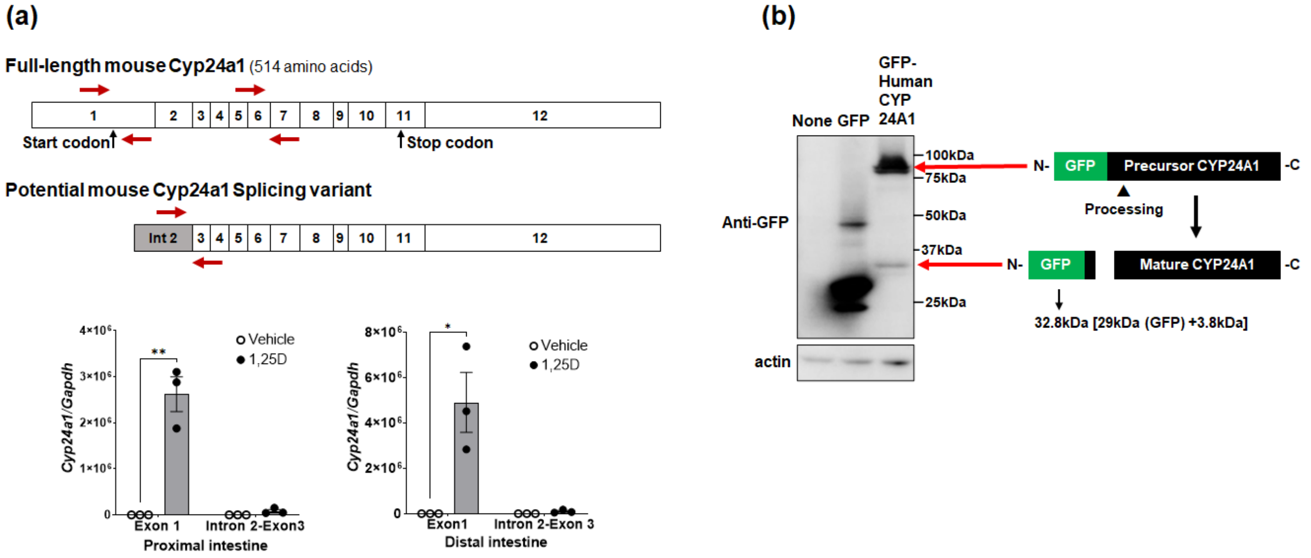

Figure S2. Mature Cyp24a1 (55kDa) in mouse is not from a splicing variant.
(a) We designed full-length specific primers (exon 1) or potential splicing variant specific primers (intron 2 - exon 3) for mouse Cyp24a1. Real-time PCR was performed with vehicle or 1,25(OH)$_2$D$_3$-treated C57BL/6J mice. The expression level in vehicle-treated mice was set to 1.0 (mean±SE; n=3; *p<0.05, **p<0.01). (b) We constructed a GFP-tagged CYP24A1 because a Flag-tagged N-fragment is too small and cannot be detected. This GFP-tagged CYP24A1 plasmid was then transfected in HEK293 cells. The total cell lysate (20 μg/lane) was separated by 15% SDS-PAGE gel. Anti-GFP detected both the full-length fusion protein (90 kDa) and the N-fragment (32.8 kDa).

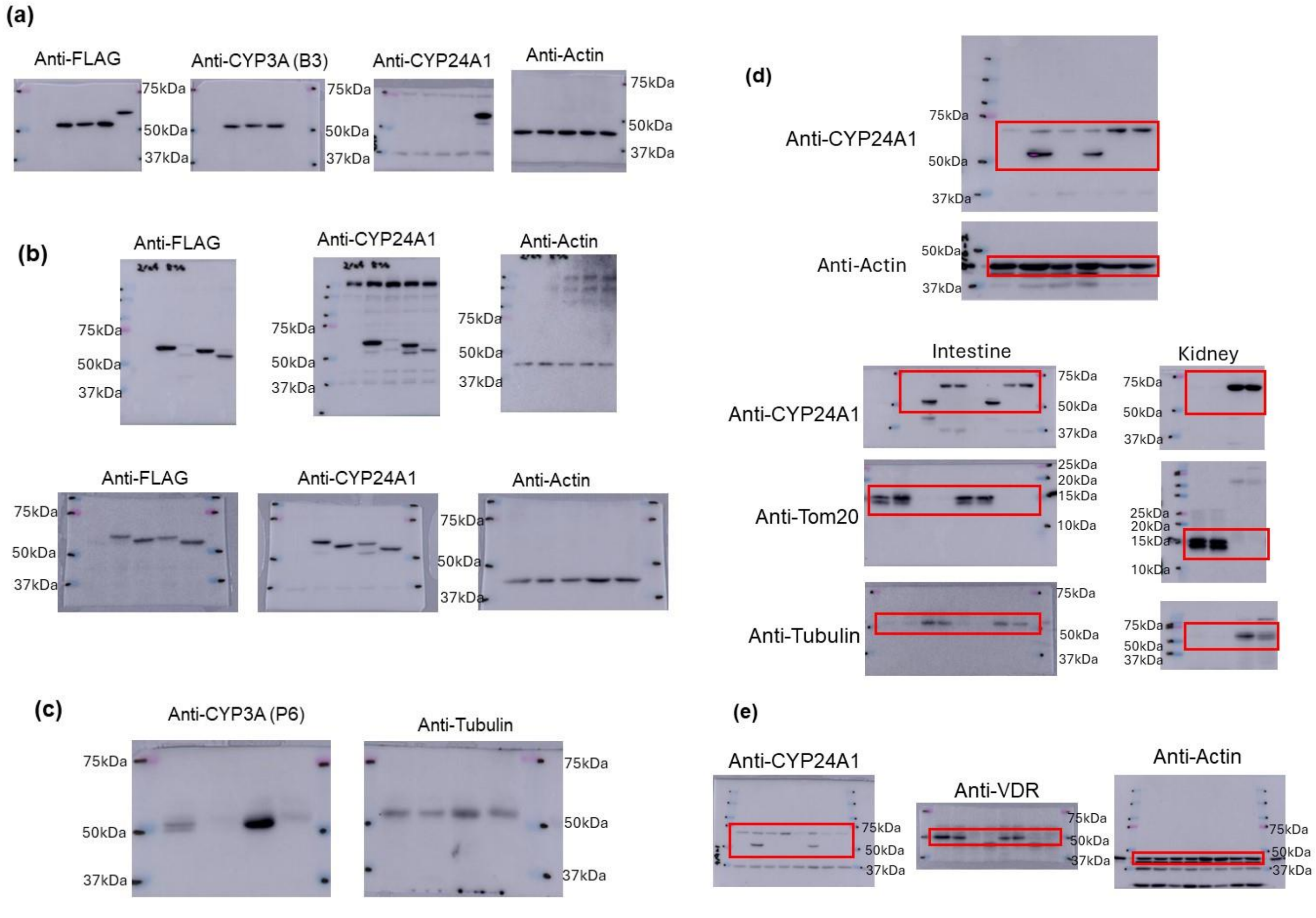

Figure S3. Raw data of western blotting.
(a) Figure 1, (b) Figure 2, (c) Figure 3, (d) Figure 4, (e) Figure 5.
